# Gut Microbiota and Their Neuroinflammatory Implications in Alzheimer’s Disease

**DOI:** 10.3390/nu10111765

**Published:** 2018-11-14

**Authors:** Vo Van Giau, Si Ying Wu, Angelo Jamerlan, Seong Soo A. An, SangYun Kim, John Hulme

**Affiliations:** 1 Department of Bionano Technology, Gachon Bionano Research Institute, Gachon University, 1342 Sungnam-daero, Seongnam-si, Gyeonggi-do 461-701, Korea; giauvvo@gmail.com (V.V.G.); wusiying524@gmail.com (S.Y.W.); angelojamerlan@gmail.com (A.J.); 2 Department of Neurology, Seoul National University College of Medicine & Neurocognitive Behavior Center, Seoul National University Bundang Hospital, Seoul 100-011, Korea; neuroksy@snu.ac.kr

**Keywords:** gut microbiota, MGB axis, germ-free animal, probiotic, neurodegenerative diseases, Alzheimer’s disease

## Abstract

The bidirectional communication between the central nervous system (CNS) and the gut microbiota plays a pivotal role in human health. Increasing numbers of studies suggest that the gut microbiota can influence the brain and behavior of patients. Various metabolites secreted by the gut microbiota can affect the cognitive ability of patients diagnosed with neurodegenerative diseases. Nearly one in every ten Korean senior citizens suffers from Alzheimer’s disease (AD), the most common form of dementia. This review highlights the impact of metabolites from the gut microbiota on communication pathways between the brain and gut, as well as the neuroinflammatory roles they may have in AD patients. The objectives of this review are as follows: (1) to examine the role of the intestinal microbiota in homeostatic communication between the gut microbiota and the brain, termed the microbiota–gut–brain (MGB) axis; (2) to determine the underlying mechanisms of signal dysfunction; and (3) to assess the impact of signal dysfunction induced by the microbiota on AD. This review will aid in understanding the microbiota of elderly people and the neuroinflammatory roles they may have in AD.

## 1. Introduction

**Figure 1:**
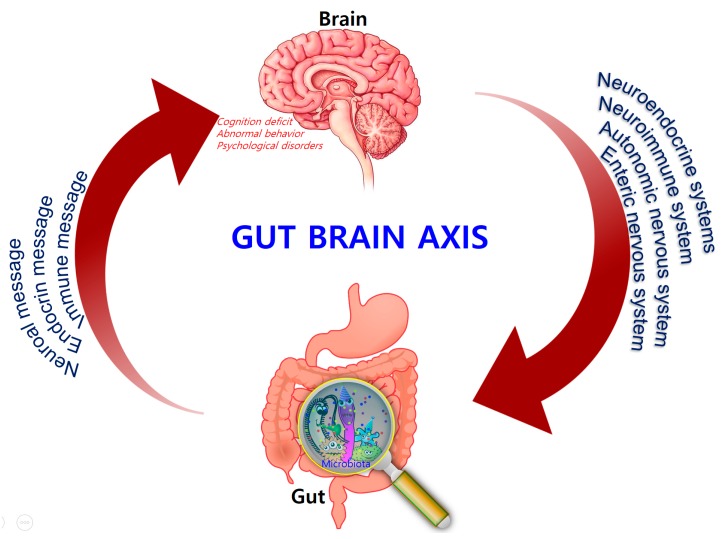
Bidirectional signaling between the gastrointestinal tract and the brain is regulated at the neural, hormonal, and immunological levels.

Comprising trillions of symbiotic microorganisms, the gut microbiota is an essential element for the maintenance of the host’s health [1,2,3]. This microbial ecosystem consists mainly of bacteria, of which most are strict anaerobes, and also fungi and viruses. The four main phyla in adults consist of *Bacteroidetes* (~48%) and *Firmicutes* (~51%), which make up the highest proportion, as well as *Proteobacteria* and *Actinobacteria*, which are found in relatively low amounts (1%) [4]. Alterations in the composition of the gut microbiota, caused by dietary changes, antibiotic exposure, and infection, lead to the loss of homeostasis, which is implicated in the development of several diseases in humans, such as colorectal cancer, metabolic syndrome, obesity, allergies, inflammatory bowel disease (IBD), type 2 diabetes, heart failure, and neurodegenerative disorders [5,6,7,8]. Recent evidence points to a causative link between pathogens and changes in the intestinal microbiota composition, along with inflammatory changes in various tissues and organs including brain tissue [7]. Hence, gut microbes may alter levels of neurotransmitter-related metabolites, affecting gut-to-brain communication and/or altering brain function (Figure 1).

Alzheimer’s disease (AD) is a neurodegenerative disorder characterized by a progressive loss of memory, language, and cognitive ability. According to the classical “amyloid cascade” model, the disease results from the over production of amyloid-beta peptide (Aβ), following the disruption of homeostatic mechanisms which regulate the proteolytic cleavage of the amyloid precursor protein (APP). Amyloids associated with AD consist largely of perivascular amyloid enriched in the 42-amino-acid Aβ42 peptide. Aβ42 is thought to initiate the neuroinflammatory process characteristic of AD pathology [8]. Plaque formation is also a natural response to infection by trapping invading microorganisms, further contributing to the collateral damage of healthy tissue that results from neuroinflammation. However, recent work suggests that the cascade model does not fully explain AD pathogenesis [9] and that alterations in the gut microbiome may play a significant role in the progression of the disease [10].

Over the last decade, several publications demonstrated the regulatory influence of the gut microbiota on the innate and adaptive immune response, as well as the importance of the interactions between the endogenous microbiota and the host’s central nervous system (CNS) [11,12]. Given the unsuccessful AD treatments employed so far, gut modification or recondition strategies started attracting the attention of the scientific community regarding the pathogenesis of CNS diseases, with emphasis on Alzheimer’s and Parkinson’s diseases [13]. In this review, we examine the gut microbiota and its interaction with the CNS, as well as its modulatory role in the neuroinflammatory process in Alzheimer’s disease.

## 2. The Intestinal Microbiota and Homeostasis

Early colonization of certain enterotypes can have a long-lasting influence on the health status of the host [14,15,16]. Human microbial colonization begins at birth. Infants born vaginally are initially colonized with microbial colonies that have a maternal signature (enriched in *Lactobacillus* and *Prevotella* spp.), while those delivered by caesarean section harbor colonies that more closely resemble the skin microbiota (enriched in *Staphylococcus* and *Propionibacterium* spp.). The microbiota then diversifies over the first few weeks of life to form a complex, anaerobe-dominated microbial community [17]. At the same time, the hypothalamic–pituitary–adrenal (HPA) axis becomes activated, which has an impact on the enteric nervous system (ENS) that innervates the gastrointestinal tract (GIT). Finally, the human gut microbiota rapidly expands and reaches an adult-like stage by three years of age. Shifts from *Bifidobacterium* to *Clostridia* and *Bacteriodetes* occur as the host develops from an infant into an adult [18]. Reductions in the level of *Faecalibacterium prauznitzii* and its anti-inflammatory relatives occur as young adults mature [6]. The composition of the microbiota is altered throughout the lifespan and is dependent on dietary and environmental factors, disease state, and other factors. In addition, several strategies suggest that *Porphyromonas gingivalis* causes an inflammatory response in the liver through the increased expression of pro-inflammatory cytokines tumor necrosis factor alpha (TNF-α) and interleukin 6 (IL-6), as well as fat storage-inducing transmembrane protein 2 (Fitm2) and perilipin 2 (Plin2), associated with lipid droplet formation, which subsequently increases neuroinflammation and causes neurodegenerative changes and AD [12,19,20].

The microbiota has long been known to play a relevant role in the health of the host. It can help break down certain nutrients, which can then be metabolized by host cells, and some of these products are involved in neural function. As such, gut bacteria produce amino acids (i.e., gamma-amino butyric acid (GABA) and tryptophan) [21,22], and monoamines (i.e., serotonin, histamine, and dopamine), which play a significant role in the brain as neurotransmitters, or as neurotransmitter precursors [23,24]. These neuroactive products can target the CNS via the blood stream and can also influence neurons in the ENS.

In the homeostatic state, a healthy GIT has a normal and stable commensal intestinal microbiota. This provides the host with nutrition and energy through the production of vitamins [25,26,27], aids in the maintenance of intestinal epithelial barrier integrity, aids in resistance to pathogens, and plays a role in the metabolic and immune systems [28,29,30]. Furthermore, the idea that the interplay between the gut and brain can modulate behavior is emerging as an exciting concept in health and disease. The interaction between them forms an essential relationship for maintaining GI homeostasis. These essential communication pathways between the immune system and the CNS depend on neurotransmitters, neuropeptides, cytokines, hormones, and growth factors, amongst other things. Experimental evidence suggests that the gut microbiota can alter levels of circulating cytokines, which in turn can have a significant effect on several brain functions [31]. Moreover, both the afferent branch of the vagus nerve and modulation of systemic tryptophan, the precursor of the neurotransmitter serotonin, are highly implicated in relaying messages from the gut microbiota to the brain [32]. In addition, an imbalance in the gut microbiota can result in diseases such as inflammatory bowel disease (IBD), irritable bowel syndrome (IBS) [33,34], gastroenteric infections [35], allergic diseases, diabetes, and metabolic syndromes [36,37,38].

## 3. The Microbiota–Gut–Brain (MGB) Axis

In the 1880s, William James and Carl Lange first introduced the concept that bidirectional communication between the CNS and intestinal organs plays a role in emotional regulation [38,39]. Forty years later, the idea that the brain plays an important role in regulating GI function was developed by Walter Cannon [40]. A number of rodent studies also showed that the gut–brain axis is a focus of research in different fields, ranging from basic microbiology to translational applications. Consequently, the potential involvement of the gut microbiota in brain function emerged. This involvement pertains to the core microbiome, distinct enterotypes, and age-related shifts in composition, which are harmful to health [41]. The concept of the MGB axis is well established. The neuroendocrine and neuroimmune systems, in addition to the sympathetic and parasympathetic arms of the autonomic nervous system (ANS) and the ENS, are key pathways in gut–brain communication. Although the exact mechanisms mediating gut–brain interactions are not fully understood, they were suggested to involve endocrine, immune, and neural pathways (vagus nerve and enteric nervous system), leading to possible alteration in AD patients or aggravating inflammation.

The concept has now expanded and has become a quickly evolving area of research that led to convergence of research efforts in the fields of neuroscience, psychiatry, gastroenterology, and microbiology—disciplines that were previously considered to have distinct and separate research objectives and focuses.

Studies investigating the effects of intestinal microbiota composition on brain function predominantly involve animal models of behavioral disorders such as anxiety, depression, and cognitive dysfunction. Accumulating evidence suggests that the composition of the gut microbiota may also have a role in several other metabolic conditions involving the CNS [42,43]. For instance, when confronted with stress, the brain may alter the composition of the gut microbiota through the HPA axis, which can regulate cortisol secretion and effect immune cell activity, both locally in the gut and systemically. Other experiments indicate that the gut microbiota and probiotic agents can alter levels of circulating cytokines, which in turn can have a significant effect on some brain functions [31,43]. In addition, both the afferent branch of the vagus nerve and modulation of systemic tryptophan are strongly implicated in relaying signals from the gut to the brain [44,45].

The MGB axis is vital for maintaining gut homeostasis. Dysregulation of the MGB axis was implicated in various disease states, the most common being chronic functional GI disorders such as IBS, which can induce depression and can also result in decreased cognitive function [46,47]. The work done by Bravo and Tillisch et al. implicated microbiota–brain signaling in alterations of resting brain activity in key circuits involved in pain, emotion, and cognition [48]. Researchers in this area are increasingly giving recognition to the microbiota itself as being an active and highly influential contributing factor in this bidirectional communication network.

## 4. Disrupting Microbiota Effects on Brain and Behavior

The gut microbiota has become a focus of studies on the brain and behavior. Alterations in the gut microbiota can modulate the peripheral and central nervous systems, resulting in altered brain function, which gives further evidence for the existence of the MGB axis. Early studies in humans demonstrate that altering the microbiota with beneficial bacteria or probiotics can lead to changes in brain function, as well as subjective reports of mood. Experimental approaches in MGB-axis research include the use of germ-free animals, animals with pathogenic bacterial infections, and animals exposed to probiotic agents or antibiotics [49].

Germ-free animal studies are conducted in animals born and reared in sterile conditions, eliminating the opportunity for postnatal colonization of the GIT. Thus, research conducted in germ-free animals highlighted the important role the gut microbiota plays in the development of both physiologic and metabolic abnormalities [49]. When compared with conventional animals, germ-free animals exhibit abnormal gastrointestinal motility, increased expression of genes encoding transporters throughout the gut, and an altered response to inflammatory pain [50,51,52]. In addition, germ-free animals have an immature and dysregulated immune system, with abnormal immunoglobulin A (IgA) production [52,53,54,55,56] and decreased numbers of intestinal mast cells [57].

The absence of gut bacteria during development affects the HPA axis [58], which has a significant role in the stress response. Studies in germ-free animals clearly demonstrate a relationship between the gut microbiota and stress- and anxiety-related behaviors. Further studies should concentrate on the influence of time, sex, strain, and other species factors on this relationship.

Infection studies are used to assess the effects of pathogenic bacteria on brain and behavior which are mediated largely through activation of the immune system [59]. Lyte et al. showed that infection-induced c-Fos expression, a marker for cellular activation, and abnormal anxiety-like behaviors can be triggered by stress [58]. Bercik et al. sought to determine the effects of chronic gut inflammation on behavior. In this study, mice were infected with *Trichuris muris*, which is very closely related to the human parasite *T. trichiura*, and alterations in anxiety-like behavior and brain-derived neurotrophic factor (BDNF) expression were examined [60]. Mice were either treated with anti-inflammatory agents, the probiotic *B. longum*, or vagotomy. All treatments normalized the infection-induced behavioral changes; however, the microbiota may communicate with the brain via several routes.

Probiotics are considered to be a novel and safe way of maintaining a healthy intestinal microbiota by producing antimicrobial agents or metabolic compounds that suppress the growth of other microorganisms [61,62] or by competing with other intestinal microbes for receptors and binding sites on the intestinal mucosa [63]. The use of probiotics was shown to improve behaviors associated with stress-related psychiatric conditions and induce neuronal plasticity. Consequently, they appear to protect the brain from stress-induced insults to the brain circuitry. For instance, probiotic-induced promotion of neurogenesis, such as increases in BDNF, in the hippocampus normalizes the abnormal response of the HPA axis. Increases in BDNF can downregulate the expression of inflammatory cytokines, decrease oxidative stress, and also improve the nutritional state [64]. A number of studies also showed that most probiotics from the *Lactobacilli* family have the potential to reduce corticosterone levels and colonic permeability, and can also affect the excitability of enteric neurons and colonic motility in the maternal separation rat model of early life stress [65]. Certain probiotic strains might be useful for improving cognitive and emotional aspects of mental health disorders. Furthermore, combining probiotic treatment with psychopharmacology may allow for the administration of lower doses of pharmacologically active compounds, thus improving their safety and reducing their toxic side effects. However, probiotic therapies have limitations, including a poor ability to establish a stable population within the recipient. Furthermore, in many instances, pathogenesis may be contributed to by broad functions conserved across many different species, such as the ability to produce metabolites that are immunomodulatory, or that directly influence brain activity. Here, it may be the absence of suitable drivers of beneficial behavior that is limiting, rather than the absence of microbes capable of exhibiting them. In such instances, the broad-scale alteration of the microbiome using selective dietary microbial growth substrates or prebiotics may be more appropriate and result in longer-lasting change. For example, consumption of fructooligosaccharides or a nondigestible galactooligosaccharide formulation (BGOS) elevates BDNF levels and *N*-methyl-d-aspartate receptor (NMDAR) subunit expression in rats [60].

Antibiotics are normally used to remove or prevent bacterial colonization in the human body, without targeting specific types of bacteria. As a result, broad-spectrum antibiotics can greatly affect the composition of the gut microbiota, reduce the bio-diversity of the fecal microbiota, and delay colonization for a long period after administration. A number of studies showed that different antibiotic treatments result in short- and/or long-term changes in the intestinal microbiota in both humans and animals. The actions of several antibiotics on gut microbial diversity are outlined in Table 1 [66,67,68,69,70,71,72,73].

## 5. Microbiota and Neurodegenerative Diseases

Amounting evidence suggests that gut microbiota plays an important role in the development of brain, and that there is a bidirectional relationship between the brain, gut, and the bacteria within the gut which is referred as the brain–gut–microbiome axis [74]. The microbiota can affect regulation of the MGB axis via immunological, neuroendocrine, and direct neural mechanisms. The gut microbiota is known to increase local and systemic inflammation due to lipopolysaccharide (LPS) from pathogenic bacteria and the synthesis of pro-inflammatory cytokines [75,76]. These microorganisms are able to produce neurotransmitters and neuromodulators, such as short-chain fatty acids (SCFAs), biogenic amines (e.g., histamine), and other amino-acid-derived metabolites such as serotonin [77] or GABA [78,79]. In addition, bacterial enzymes may also synthesize neurotoxic metabolites such as d-lactic acid and ammonia [80]. Signaling molecules secreted by the gut microbiota are transferred via the lymphatic and systemic circulation throughout the CNS where they then affect behavior and modulate brain plasticity and cognitive function [81]. This implicates the importance of the gut microbiota in the development and function of the CNS, and in the pathophysiology of chronic brain diseases [80]. Microbiome species and their secretory products are extremely powerful pro-inflammatory and innate-immune activators in the host [23,82,83,84,85,86,87,88,89,90].

A recent study provided additional evidence of the role of molecules secreted by gut microbiota in the brain inflammatory cascade. Two kinds of immune cells in the CNS were reported—the microglia and astrocytes. They communicate through the use of vascular endothelial growth factor B (VEGF-B) and transforming growth factor alpha (TGF-α) to regulate neuroinflammation [91]. This communication is made possible through a ligand-activated transcription factor called aryl hydrocarbon receptor (AHR), which was previously thought to have an exclusive role in processing environmental toxins. In this study, experimental autoimmune encephalitis (EAE) was induced in genetically engineered mice with AHR, which can be easily deleted in microglia (except brain cells and other immune cells) through drug treatment. Elimination of microglial AHR significantly exacerbated EAE, but did not affect immune responses outside the CNS. This suggested that AHR activation in microglia inhibits inflammation in the CNS. The study also demonstrated the direct involvement of AHR in the expression of TGF-α and VEGF-B. Additional in vivo and in vitro analyses of these cytokines showed their role in regulating pro-inflammatory reactivity of astrocytes. TGF-α reduces astrocyte inflammatory responses in EAE, and its expression in microglia is impeded by AHR deletion. In contrast, VEGF-B increases astrocyte inflammatory responses in EAE, and its expression is enhanced by AHR deletion [91]. In addition, CNS inflammation was reduced in antibiotic-treated mice by supplementation with the tryptophan metabolites indole, indoxyl-3-sulfate (I3S), indole-3-propionic acid (IPA), and indole-3-aldehyde (IAld), or the bacterial enzyme tryptophanase [92]. It is worth noting that several of the aforementioned metabolites are proven quorum antagonists [93,94]. In addition to metabolites bacterial peptides readily cross the blood-brain barrier as well [95], which begs the question do these quorum molecules induce significant aberrations in communication between specific neural structures of the brain (dopaminergic (DA) neurons) [96].

The connection between the kind of gut microbiota and AD pathology was shown in a study that used transgenic mouse models. Harach et al. observed a significant shift in the diversity of gut microbiota of APP transgenic mice with that of non-transgenic wild-type mice through sequencing of 16S ribosomal RNA (rRNA) from their fecal samples [97]. Germ-free APP transgenic mice were generated, in which a significant decrease in cerebral Aβ pathology was observed when compared to control mice with intestinal microbiota. Interestingly, an increase in cerebral Aβ pathology was observed in germ-free APP transgenic mice when these were colonized with gut microbiota acquired from conventionally raised APP transgenic mice [98].

The specific role of gut microbiota in modulating neuro-immune functions well beyond the gastrointestinal tract may constitute an important influence on the process of neurodegeneration, especially that associated with AD [41,99]. Bacteria and fungi, as a component of human gut microbiota, secrete amyloid protein in CNS, resulting in Aβ accumulation and increased risk of AD [100,101,102,103,104,105,106,107,108]. Limiting amyloid accumulation via periodic fasting-mimicking diet (FMD) was proposed as an alternative approach to increase the protection of multiple systems in mice and possibly humans [109]. Preliminary results of the randomized, parallel-group, three-arm pilot trial to test the feasibility of prolonged fasting and ketogenic diet in relapsing-remitting multiple sclerosis (IGEL) (clinicaltrials.gov identifier: NCT01538355) showed a reduction in the number of autoimmune lymphocytes, an increase in the β-hydroxybutyrate concentrations in plasma, and a reduction in relapses and expanded disability status scale in the FMD and ketogenic diet groups. FMD ameliorates CNS damage and behavioral outcomes in EAE mice by increasing the corticosterone levels and regulatory T (Treg) cell numbers, and reducing the levels of pro-inflammatory cytokines, T-helper 1 (Th1) and Th17 cells, and antigen-presenting cells (APCs) [110]. These effects are possibly related to an arrest of B- and T-cell development and concomitant selective localization of mature B- and T-cell numbers in the bone marrow seen in fasting mice [111].

Recent studies suggest that gut microbiota is altered in AD patients and may be involved in the pathogenesis of AD. Several bacteria taxa in AD patients were different from those in controls at taxonomic levels, such as Bacteroides Actinobacteria, Ruminococcus, Lachnospiraceae, and Selenomonadales, from 43 AD patients using 16S ribosomal RNA sequencing [112]. In addition, many bacteria are capable of synthesizing and releasing many neurotransmitters and neuromodulators themselves, as well as neuropeptides from enteroendocrine cells, indicating a possible involvement of gut microbiota in the development of AD pathology (Table 2) [23,86,87,88,89,90,97,99,112,113,114].

## 6. The Role of Inflammation in Alzheimer’s Disease

Inflammatory reactions could be both beneficial and detrimental to the brain, depending on strengths of their activation in various stages of neurodegeneration [112]. Regulation of immuno-inflammatory control is one of the relevant processes involved in the pathogenesis of neurodegenerative disorders. AD shares several common properties with other neurodegenerative disorders, such as accumulation of misfolded proteins (Aβ) and hyperphosphorylated tau, evidence for a prion-like spread of pathology with misfolded proteins and neuroinflammation [9]. The pro-inflammatory gut microbiota dysbiosis in AD patients could trigger inflammation-induced formation and aggregation of cerebral amyloid-β, proving to be an effective strategy for preventing or reducing the risk of AD [115]. Bacterial strains known to produce functional extracellular amyloid fibers are *Escherichia coli*, *Salmonella enterica*, *Salmonella typhimurium*, *Bacillus subtilis*, *Mycobacterium tuberculosis*, and *Staphylococcus aureus* [115]. For instance, *E. coli* endotoxin was shown to induce the formation of Aβ fibrils in vitro, implying their involvement in AD pathogenesis [115]. Numerous studies established a role for neuroinflammation in AD pathology [116]. The amyloid cascade hypothesis is the prevailing theory that the presence of Aβ plaques primarily results in synaptic dysfunction and dementia in AD. However, this was challenged by Honig et al. when they showed that treatment of AD patients every week for four weeks with 400 mg of solanezumab, which clears soluble Aβ from the brain, did not significantly affect cognitive decline [117,118]. Moreover, the drug efficacy can be affected by some factors such as the presence of the blood–brain barrier. Additional research presented evidence of other key players of neuroinflammation in the CNS, such as microglia, astrocytes, the complement system, and cytokines. More specifically, cytokines play a central role in neuroinflammation [119,120]. Several studies demonstrated abnormally elevated levels of inflammatory cytokines, such as interleukin IL-1β and TNF, in AD patients. Activated microglia were shown to be involved in the secretion of pro-inflammatory cytokines such as IL-1, IL-6, TNF-α, and TGF-β, thereby contributing to the progress of neurological disorders [82,83,91,121]. In AD particularly, proper cessation of inflammation is compromised due to the constant deposition of Aβ plaques, as well as the positive feedback loop between inflammation and amyloid precursor protein (APP) processing [118]. In addition, studies on AD pathogenesis showed that microglia produce Aβ, which by itself is pro-inflammatory and causes activation of several inflammatory mediators [12,122,123,124,125].

**Figure 2:**
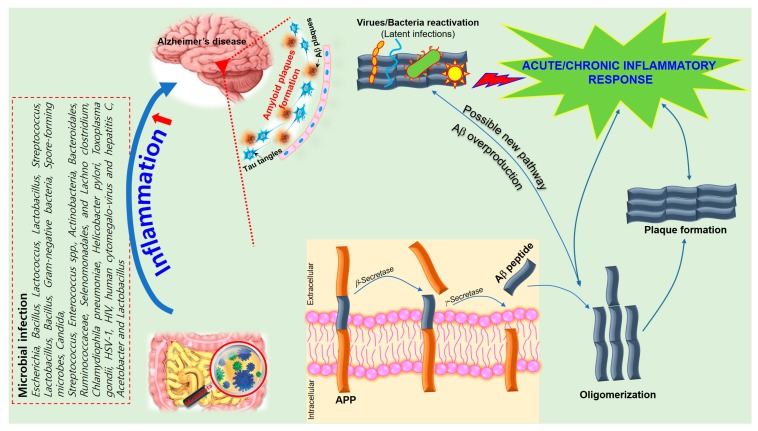
A schematic of the hypothetical chain of events via which brain infection may lead to pathological amyloid-β peptide (Aβ) plaque formation in the brain. The amyloid precursor protein (APP) is processed by secretases into different peptides, including Aβ. The gut microbiota plays a significant role in the development of Alzheimer’s disease (AD) since Aβ functions as an antimicrobial peptide via oligomerization and plaque formation, trapping invading microorganisms, including bacteria, fungi, viruses, and protist parasites (detailed in Table 2). Aβ plaque formation in response to infection could result in a neuroinflammatory effect of microbiota on AD and neurodegeneration due to collateral damage in plaque-surrounding tissue.

Activated astrocytes are supportive cells that provide trophic and metabolic maintenance for neurons and are also influential in neuroinflammation in AD. Changes in astrocyte morphology, gene expression, protein composition, and activity can be observed in AD, which in turn can compromise astrocyte function [126]. Several transgenic mouse models showed that activated astrocytes accumulate in the brain before any plaque or tangle pathology can be observed [119], suggesting that astrocyte activation may be involved in AD pathogenesis. Astrocytes outnumber microglia in the brain and have a greater influence on long-term neuroinflammation. They also secrete pro-inflammatory cytokines and chemokines to process and clear away accumulated Aβ. The additional deposition of Aβ then results in a positive feedback loop that furthers astrocyte activation resulting in the release of more pro-inflammatory factors [118]. Thus, it is essential to further explore the key inflammatory components in AD pathogenesis in order to develop better treatments for these disorders (Figure 2).

## 7. Neuroinflammatory Effects of Microbiota on AD

In AD, the cerebellum is replete of microglia in areas of amyloid deposition, and cerebellar volume is reduced [127]. Molecular layer gliosis and atrophy in the vermis is also severe. Loss of Purkinje neurons occurs in the vermis, cerebellar hemispheres, and the inferior olivary nucleus [128,129]. Atrophy of the molecular layer by 24% and the granular layer by 22% correlates with a decrease in Purkinje cell numbers [130]. This might be related to BDNF, which is essential for the maintenance and survival of neurons, and which has pleiotropic effects on neuronal development, differentiation, synaptogenesis, and the synaptic plasticity that underlies neuronal circuit formation and cognition. Decreased amounts of BDNF were found in AD brains [131,132]. Interestingly, mice deficient in BDNF have altered development of GI-tract innervations [133,134]. BDNF expression was found to be reduced in the hippocampus and cortex of germ-free mice, and reduced expression of BDNF was found to be specifically associated with increased anxiety and progressive cognitive dysfunction [91,135].

Pro-inflammatory cytokines are already known to enhance APP expression, upregulate β-secretase messenger RNA (mRNA), and increase Aβ formation in the hippocampus. Similarly, bacterial amyloid is recognized as a pathogen-associated molecular pattern that can cause activation of Toll-like receptor-2 (TLR2), which was also reported to induce *Notch1* upregulation and activation of microglia, and may enhance processes leading to the development of AD or Parkinson’s disease (PD) [135]. Functional bacterial amyloid may be the source of (1) misfolding of neuronal proteins through cross-seeding and (2) activation of the innate immune system and priming of neuroinflammation. The unique structure of this foreign amyloid might induce specific misfolding patterns which could be responsible for the variety of phenotypes in neurodegenerative disorders. APP expression may also influence the kind of microbiota that thrive in the gut. Reduction in populations of Firmicutes, Verrucomicrobia, Proteobacteria, and Actinobacteria were observed in the gut of conventionally raised amyloid precursor protein (APP) gene with Swedish mutation and presenilin 1 gene (PS1) with deletion of exon 9 (APPPS1) transgenic mice when compared to normal wild-type controls, both aged eight months [98]. In contrast, an increase in Bacteroidetes and Tenericutes phyla were observed [98]. Subsequently, germ-free generated APPPS1 mice showed decreased levels of cerebral Aβ42 when compared to conventionally raised APPPS1 mice, further supporting the possible influence of gut microbiota on APP expression [98].

Chromogranin A (CHGA) is a neuroinflammatory factor which is frequently present in AD senile plaques associated with microglial activation [136]. Wu and Nakanishi found that CHGA activated both nuclear factor kappa B (NF-κB) and pro-caspase-1, while Aβ only activated pro-caspase-1. For activation of pro-caspase-1, both CHGA and Aβ require the enzymatic activity of cathepsin B (CatB). In the AD brain, highly activated microglia showed intense immunoreactivity for CatB and IL-1β, and they surround CHGA-positive plaques more frequently than Aβ-positive plaques. This suggests different pathways for CHGA- and Aβ-induced microglial production of IL-1β, which may help us gain a better understanding of the pathological significance of neuroinflammation in AD [137].

In addition, chronic gut inflammation may increase the breakdown of blood–brain barrier, LPS permeability, and the generation of pro-inflammatory cytokines. Elevated levels of LPS were found in the plasma of AD patients compared to healthy controls [82]. In an animal study, peripheral administration of LPS led to neuroinflammation with an increase in levels of IL-6. Moreover, overexpression of IL-1β resulted in a robust increase in tau phosphorylation in the triple transgenic mouse model of AD [121,135]. Furthermore, recent studies suggested that increased concentrations of circulating LPS, promoted by changes in intestinal permeability, may play a pivotal role in insulin resistance. Neuronal insulin resistance can increase the risk of developing AD, and insulin treatment may enhance memory function. Carvalho et al. found that antibiotic treatment greatly modifies the gut microbiota by reducing levels of Bacteroidetes and Firmicutes and circulating LPS levels. This modulation consequently improved glucose and insulin tolerance and activity in metabolically active tissues [138].

Furthermore, Villaran and co-workers reported that peripheral inflammation in the form of dextran sodium sulfate (DSS)-induced colitis can aggravate LPS-induced neuroinflammation and neurodegeneration as shown by increased mRNA transcripts of TNF-α, inducible nitric oxide synthase (iNOS), and IL-6 in the midbrain [4]. Interestingly, even rats with colitis alone, which received no midbrain injection of LPS, showed increased levels of TNF-α, iNOS, and IL-6 mRNA in the midbrain. Other studies also demonstrated that recurring systemic infections can increase the probability of developing multiple sclerosis (MS), AD or PD [139]. Table 2 listed the effects of some particular gut microbiota and their corresponding metabolites on nervous system function. Also included are endotoxins produced by Gram-negative bacteria, which further impact neuroinflammation through inflammatory cytokines in conditions like obesity, diabetes, and possibly AD. LPS is also a structural component of Gram-negative bacterial membranes in the gut. When the bacteria die, LPS is shed from their membranes and floats freely in the lumen as a bacterial endotoxin. LPS is harmless and is isolated from the bloodstream if the intestinal lining is intact. However, damage to the lining can cause LPS to penetrate and cause low-grade inflammation. LPS from the intestinal tract was found to be abundant in the neocortex and hippocampus of AD-affected brains [140].

Hence, the microbiota is closely related to neurological dysfunction and plays a significant role in neuroinflammation through the secretion of pro-inflammatory cytokines. Changes in the homeostatic state of the microbiota leads to increased intestinal permeability, which may promote the translocation of bacteria and endotoxins across the epithelial barrier, inducing an immunological response associated with the production of pro-inflammatory cytokines. The activation of both enteric neurons and glial cells may result in various neurological disorders [139].

## 8. Conclusions

This review described the importance of the MGB axis and the bidirectional communication between the gut and brain in regulating the health of the host. A healthy GIT in a homeostatic state has a normal and stable commensal intestinal microbiota and provides the host with nutrition and energy by producing vitamins. Braak and colleagues hypothesized that disease begins in the gut and spreads from the gut to the brain via the MGB axis. Gut-related inflammation is essential in provoking endotoxemia, systemic inflammation, and neuroinflammation, which can contribute to neurodevelopmental disorders and the onset of psychiatric illness in later life.

Previous studies provided considerable evidence of the effect of gut microbiota on the CNS, as well as its neuroinflammatory role in neurodegenerative diseases like AD. The production of amino acids by gut microbiota, which serve as components for neurotransmitters, can influence neurons both in the CNS and ENS. The kind of gut microbiota also plays a role in AD pathology as shown by cross-colonization experiments using germ-free and control APP transgenic mice. Pro-inflammatory cytokines secreted by microglia and astrocytes, and continuous Aβ deposition create a positive feedback loop that furthers neurodegeneration. This is especially made more significant by a dysregulated MGB axis, as well as chronic gut inflammation, which increases intestinal permeability, leading to the release of more pro-inflammatory factors in the CNS. Thus, it is essential to direct future studies on cell signaling and cytokine regulation to suppress neuroinflammation in AD.

## Figures and Tables

**Table 1:** Antibiotic treatment effects on the microbiota (both human and animals).

Antibiotics	Dosage	Subjects	Changes in Microbial Composition	Type and Duration of Study	Reference
Ciprofloxacin	500 mg, 3× per day for 5 days	Healthy adults	↓Clostridiales	Diversity 8 months	[14]
Amoxicillin	375 mg, 3× per day for 5 days	Healthy adults	↓*Bifidobacterium* ↑Enterobacteriaceae ↑Metabolic dysfunction	Parallel intervention 26 days	[66]
Amoxicillin	60 mg/mL 8–11 days	Rats	↑*Proteobacteria* ↑Haptoglobin levels ↓Diversity Index	Intestinal permeability 8 days	[67]
Vancomycin	500 mg, 3× per day for 7 days	Male adults. Metabolic syndrome	↓Gram-positive bacteria (Firmicutes) ↑Gram-negative bacteria (Proteobacteria) ↓Peripheral insulin sensitivity	Single blinded randomized controlled 1 week	[68]
Vancomycin	0.2 mg/mL for 8 weeks	NOD mice	↑*Escherichia*, ↑*Lactobacillus* ↑*Sutterella*	Disease (type 1 diabetes) 40 weeks	[69]
Metronidazole	500 mg/L for 4 weeks	Mice	↓Alpha diversity ↓*Bacteroidetes* ↑*Akkermansia muciniphila*	Glucose metabolism 4 weeks	[70]
Ampicillin and Gentamicin	Parenteral treatment (within 48 h of birth)	Newborn babies	↑Proteobacteria ↓Actinobacteria and *Lactobacillus*	Developmental 2 months	[71]
Cefalexin	50 mg/kg, 4× per day for 4 days	Newborn babies	↑*Enterococcus* spp. and ↑Enterobacteriaceae	Developmental 7 days	[72]
Clindamycin	150 mg, 4× per day for 7 days	Healthy adults	↑Frequencies of highly antibiotic-resistant clones ↓Bacteroides diversity	Diversity 24 months	[73]
F-quinolones and β-lactams Combination	Variable dose 7 days	Admitted patients	↑Bacteroidetes ↓25% microbial taxa	Infection 1 week	[74]
Clarithromycin Metronidazole Combination	400 + 250 mg, 2× per day for 7 days	*Helicobacter pylori*-infected adults	↓Diversity, particularly Actinobacteria in faeces ↑*ermB* gene levels	Diversity 6 months	[75]

Note: ↓, decrease; ↑, Increase; NOD, non-obese diabetic.

**Table 2:** Potential links between gut microbiota and the development of Alzheimer’s disease (AD).

Gut Microbiota	Metabolite Product	Effects on Nervous System Function	References
*Escherichia*, *Bacillus*, *Lactococcus*, *Lactobacillus*, *Streptococcus*	Dopamine	System activity, Parkinson’s disease, AD, and depression-related	[86,87,88]
*Lactobacillus*, *Bacillus*	Acetylcholine	Acting on neurotransmitters in the central and peripheral nervous systems, and cognitive function, particularly closely related to learning and memory	[89]
*Lactobacillus*, *Lactococcus*, *Streptococcus*, *Enterococcus*	Histamine	Regulate neurotransmitters; sleep and cognitive function related	[23,90]
Gram-negative bacteria	Endotoxin	Induce inflammation, release large amounts of inflammatory cytokines (TNF-α, IL-6, and IL-8, etc.), obesity, IR, and diabetes, and are closely related to the occurrence of AD	[97,113]
Actinobacteria, Bacteroidales, Ruminococcaceae, Selenomonadales, and *Lachnoclostridium*	Neural, endocrine, and immune pathways	Impairment and brain amyloidosis, neuroinflammation	[112]
*Chlamydiophila pneumoniae*, *Helicobacter pylori*, *Toxoplasma gondii*	Pro-inflammatory cytokines and induction of oxidative	The presence of the bacteria in astrocytes, microglia, neurons, and in infected cells close to senile plaques and intracellular neurofibrillary tangles	[101,102,103,104]
	stress, immune regulation, and apoptosis		
Viruses (HSV-1, HIV, human cytomegalo-virus, and hepatitis C)	Microbiome-derived amyloids	Microbial amyloids may play a role in the homeostasis and pathology of the CNS with particular reference to AD	[105,106,107]
*Porphyromonas gingivalis*	Pro-inflammatory cytokines TNF-α, IL-6, and IL-1β	Subsequently increase neuroinflammation and cause neurodegenerative changes and AD	[19,20]
*Acetobacter* and *Lactobacillus*	Regulating acetate (short-chain fatty acids—SCFA) in *Drosophila* model	Participate in AD pathogenesis by influencing SCFA level	[99,108]

TNF-α: tumor necrosis factor alpha; IL-6: interleukin 6; IL-8: interleukin 8; CNS: central nervous system; SCFA: short-chain fatty acids; IR: Insulin resistance; HSV-1, Herpes Simplex Virus 1; HIV, Human Immunodeficiency Virus; CNS: central nervous system.
